# Current practices in managing patients with cardiac implantable electronic devices: Results of an international survey

**DOI:** 10.1016/j.hroo.2025.02.019

**Published:** 2025-03-05

**Authors:** James Allred, Amber Seiler, Mark Lyons, Paul Roberts, Angela Tsiperfal, Laura van Heel, Catherina Meijer, Emmanuelle Nicolle, David Lanctin, Eimo Martens

**Affiliations:** 1 CV Remote Solutions, Greensboro, North Carolina; 2 Cardiology and Physiology Department, Nottingham University Hospitals NHS Trust, Nottingham, United Kingdom; 3 Cardiology and Physiology Department, University Hospital Southampton NHS Foundation Trust, Southampton, United Kingdom; 4 Cardiac Electrophysiology Department, Stanford Health Care, Stanford, California; 5 Health Economics and Outcomes Research Division, Deloitte, Brussels, Belgium; 6 Cardiac Rhythm Management, Medtronic, Mounds View, Minnesota; 7 Device Therapy and Telemedicine Department, Technical University of Munich, Munich, Germany

**Keywords:** Cardiovascular implantable electronic device, Patient management, Remote monitoring, Efficiency, Survey, Quality improvement

## Abstract

**Background:**

Managing patients with cardiac implantable electronic devices (CIEDs) is increasingly complicated with expanding populations and evolving technologies. While the 2023 Heart Rhythm Society/European Heart Rhythm Association/Asia Pacific Heart Rhythm Society/Latin American Heart Rhythm Society consensus on management of remote device clinics provides recommendations, the adoption of these practices in real-world practice is unknown.

**Objective:**

This survey of device clinic staff characterized the adoption and variability of CIED follow-up practices.

**Methods:**

A Delphi panel composed of U.S. and European Union clinical experts guided the research objectives, structure, and content of the survey. Once consensus was reached, the online survey (Qualtrics) was deployed in partnership with HRS through e-mail, social media posts, and at the HRX conference. An available case analysis was performed to handle missing data.

**Results:**

A total of 471 responses were collected from 44 countries, including 310 in the United States, 88 in Europe, and 73 in other regions. Broad representation was achieved with respect to staff role, years of experience, and clinic ownership. Most (71%) respondents reported being aware of the 2023 consensus statement. While the majority (77%–83%, depending on device type) reported using hybrid in-person and remote management for patients with therapeutic CIEDs, 89% to 91% reported scheduling routine office visits at least once per year, depending on device type, respectively. Only 50% of respondents reported a hybrid approach for insertable cardiac monitor patients, with 35% reporting remote-only follow-up.

**Conclusion:**

Variable adoption was found for many practices recommended in the 2023 Heart Rhythm Society/European Heart Rhythm Association/Asia Pacific Heart Rhythm Society/Latin American Heart Rhythm Society consensus. Future research should focus on optimal implementation of recommended practices.

**Key Findings**

▪ While most respondents were reportedly aware of the 2023 Heart Rhythm Society/European Heart Rhythm Association/Asia Pacific Heart Rhythm Society/Latin American Heart Rhythm Society consensus on management of remote device clinics, meaningful disparities were found between the consensus statement recommendations and reported practices.
▪ Remote monitoring practices were more homogeneous in the United States with respect to the device types remotely monitored and the frequency of scheduled transmissions; in Europe, much more heterogeneity was observed.
▪ Despite low reported actionability of routine office visits for patients with cardiac implantable electronic devices, 90% of respondents reported scheduling routine office visits for the average pacemaker of implantable cardioverter-defibrillator patient either once or twice per year.
▪ Only a small minority of respondents reported their clinic to be engaged in ongoing performance assessment. Of those tracking clinic performance, the most common metrics tracked were predominantly measures of productivity, rather than measures of quality.

## Introduction

The growing population living with cardiac implantable electronic devices (CIEDs) has created a significant workload for cardiac device clinics, raising questions on the best follow-up practices to efficiently optimize patient outcomes. Originally, routine office visits were regularly scheduled depending on patient history, local reimbursement policies, and device type. As technology evolved, the combination of remote monitoring (RM) and face-to-face follow-up has emerged as standard of care in international guidelines since 2015.^1^ RM enables continuous monitoring and closer patient care with earlier detection of device-related and clinical events, allowing for more timely intervention leading to improved clinical outcomes and reduced healthcare utilization.^1^^,^^2^ Despite the advancements in CIED technology and expanded use of RM, patient management still presents several challenges. This includes establishing and maintaining the necessary infrastructure for efficient clinic operations, ensuring sufficient staffing levels with clearly defined roles and responsibilities for the effective management of clinic workflows, and ensuring appropriate funding to sustain clinics and incentivize an efficient healthcare practice.^2^

Although organizational aspects to optimize CIED patient follow-up are crucial, research on this topic remains limited. A time and motion workflow evaluation recently described the CIED management tasks performed and quantified the associated workload.^3^ This confirmed the complexity of patient management with significant clinical and administrative staff time across in-person office visits, remote transmission review, and other patient management tasks. However, this evaluation could not provide a broad picture of existing organizational models, the prevalence and variability of patient management practices, or staff perceptions related to patient management burden and efficiency.

The 2023 Heart Rhythm Society (HRS)/European Heart Rhythm Association (EHRA)/Asia Pacific Heart Rhythm Society (APHRS)/Latin American Heart Rhythm Society (LAHRS) expert consensus statement on practical management of the remote device clinic made recommendations on in-clinic and RM visit frequency, modalities, data interpretation, and integration into clinical practice.^2^ These recommendations provide a blueprint for standardizing CIED management, reducing disparities in care and optimizing health and economic outcomes. However, the current state and variability of practices across clinics remain unclear. Gaining further understanding on contemporary practices in CIED patient management may help to identify areas of improvement to enhance patient care and move toward the effective implementation of these recommendations. Thus, an international survey was developed to characterize contemporary practices and perceptions in patient management from the perspective of CIED device clinic staff.

## Methods

### Survey development

An expert committee of 7 clinic advisors from Europe and North America was established to guide development of the survey. A modified Delphi technique,^4^ consisting of 2 questionnaire rounds, was used to reach consensus on the research topics included and the specific questions for the survey. Preliminary research topics were broadly identified through an informal review of established literature and honed by the opinions of the expert committee. In the first Delphi round, the expert committee was asked to rate the preliminary research topics for inclusion in the survey based on a 5-point Likert scale and to provide suggestions for improvement. For the research topics that reached consensus agreement for inclusion, based on an a priori defined threshold,^5^ survey questions were drafted. These, in addition to revised research topics were shared with the expert committee for a second round of assessment. Research topics meeting the final consensus agreement, based on an a priori defined threshold,^5^ were incorporated into the survey. These included (1) follow-up practices, (2) clinic performance, (3) patient management solution adoption, and (4) staff perceptions.

The final survey questions were reviewed by the expert committee for content and face validity, then piloted with the same committee to refine and finalize the questionnaire. The online survey was programmed using Qualtrics software and hosted on a server with controlled access. Some questions were tailored to individual respondents’ previous responses through the use of conditions and display logic functions. For instance, respondents reporting “remote only” follow-up would only be asked about scheduled transmission frequencies, and not about routine office visit frequency. A copy of the survey is available in the Supplemental Materials. This research project adhered to the Helsinki declaration as revised in 2013.

### Survey distribution

The survey was administered to CIED device clinic staff between September 2023 and January 2024. Distribution of the survey was facilitated in collaboration with the HRS via email, social media posts, and during the HRX conference (September 21–23, 2023). In addition, the survey was distributed through CareLink, a proprietary management software for healthcare professionals. No formal sample size was prespecified and no compensation was offered for completion of the survey.

### Data analysis

Categorical responses from the closed-ended questions were reported as proportions. Subanalyses were performed by region, staff role, and clinic characteristics to explore the potential impact of these factors on the findings. Due to sample size limitations, regional comparisons were restricted to the United States and Europe. Appropriate statistical tests were performed using SAS 8.3 software (SAS Institute) to analyze the data and determine the statistical significance of observed differences between groups. An available case analysis approach was utilized, including all available data for each specific analysis.

## Results

### Respondent characteristics

A total of 471 survey responses were collected, with the characteristics of the respondents detailed in Table 1. The majority of respondents were from the United States (66% [n = 310]), followed by Europe (19% [n = 88]), and reported working in a hospital-owned clinic (74% [n = 347]). The most common specialties among respondents were nurse (24% [n = 115]), electrophysiologist (23% [n = 110]), and device technician (19%). Just over half of the respondents reported more than 15 years of professional experience (52% [n = 243]).

**Table 1:** Demographics and characteristics of survey respondents.

Parameter	Respondents (n = 471)
Location	
Europe	89 (19)
Asia-Pacific	31 (7)
United States	310 (66)
Americas	26 (6)
Middle East and Africa	12 (3)
Unknown	3 (1)
Specialty	
Advanced practice provider	43 (9)
Administration assistant	6 (1)
Clinic manager	42 (9)
EP	110 (23)
Nurse	115 (24)
Non-EP cardiologist	18 (4)
Physiologist	24 (5)
Device technician	91 (19)
Other	22 (5)
Years of working experience	
<5	72 (15)
5–14	158 (33)
15–20	91 (19)
≥20	152 (32)
Clinic ownership	
Independent	98 (21)
Hospital-owned	347 (74)
Other	26 (6)

Values are n (%). Note on number of responses, by country: Europe (Austria, n = 2; Belarus, n = 1; Belgium, n = 1; Bulgaria, n = 1; France, n = 4; Germany, n = 11; Greece, n = 4; Ireland, n = 4; Italy, n = 9; Netherlands, n = 4; Norway, n = 1; Poland, n = 3; Portugal, n = 8; Romania, n = 1; Serbia, n = 1; Spain, n = 5; Sweden, n = 1; Turkey, n = 5; United Kingdom, n = 24), Asia-Pacific (Afghanistan, n = 1; Australia, n = 11; Bangladesh, n = 1; China, n = 1; India, n = 7; Japan, n = 2; New Zealand, n = 2; Pakistan, n = 2; Singapore, n = 1; Vietnam, n = 3), South America (Argentina, n = 1; Brazil, n = 3; Ecuador, n = 2; El Salvador, n = 1), North America (Canada, n = 18; United States, n = 310; Mexico, n = 1), Middle East and Africa (Bahrain, n = 1; Iran, n = 1) Israel, n = 2; Jordan, n = 1; Morocco, n = 1; Saudi Arabia, n = 2; South Africa, n = 2; Turkey, n = 1; United Arab Emirates, n = 1), and unknown (n = 3).

EP, electrophysiologist.

### HRS consensus statement awareness

Out of 428 respondents who reported on guideline awareness for remote monitoring, 71% (n = 302) were aware of the 2023 HRS/EHRA/APHRS/LAHRS expert consensus statement. Managing a higher number of patients was associated with increased consensus statement awareness. In particular, the mean number of pacemaker patients managed by clinics reporting consensus statement awareness was 1742, compared with 943 for clinics reporting not being aware (*P* < .001) (Supplemental Figure 1a). Similarly, the mean number of implantable cardioverter-defibrillator (ICD) patients managed by clinics reporting consensus awareness was 875, compared with 553 patients for clinics reporting not being aware (*P* = .01). In addition, guideline awareness was more frequently reported by advanced practice providers, electrophysiologists, and physiologists (Supplemental Figure 1b).

### Patient follow-up practices

**Figure 1:**
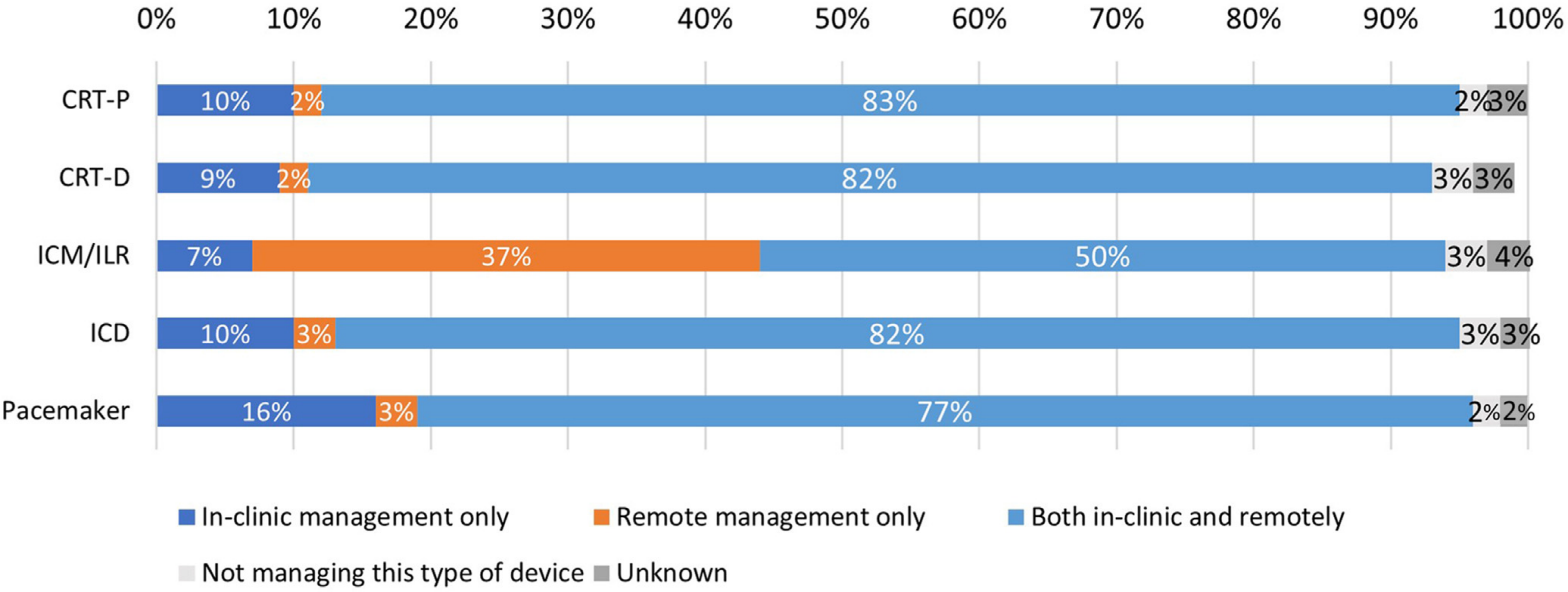
Patient follow-up approach by device type. CRT-D, cardiac resynchronization therapy defibrillator; CRT-P, cardiac resynchronization therapy pacemaker; ICD, implantable cardioverter-defibrillator; ICM, insertable cardiac monitor; ILR, implantable loop recorder.

Out of the respondents who reported on follow-up practices, hybrid management (in clinic and remote) was the most common approach for patients with a cardiac resynchronization therapy pacemaker (CRT-P) (83% [n = 276]), a cardiac resynchronization therapy defibrillator (CRT-D) (82% [n = 278]), ICD (82% [n = 274]), and pacemaker (77% [n = 258]) (Figure 1). While hybrid management was also common for patients with insertable cardiac monitors (ICMs) (50% [n = 167]), a substantial proportion of respondents reported remote-only management for these patients (37% [n = 123]). In-clinic management only was rarely reported across medical devices (pacemaker: 16% [n = 51]; CRT-P: 10% [n = 32]; ICD: 10% [n = 32]; CRT-D: 9% [n = 30]; ICM: 7% [n = 22]).

Remote monitoring—alone or combined with routine office visits—was the predominant follow-up practice for patients with a pacemaker and ICD (Table 2). Subanalyses at the geographical level revealed differences between respondents from the United States and Europe (Supplemental Table 1). In particular, 96% (n = 210) and 94% (n = 211) of respondents from the United States reported remote management as a follow-up practice for patients with a pacemaker and ICD, respectively. In comparison, only 54% (n = 33) of European respondents reported using remote management for pacemaker patients, and 75% (n = 46) for ICD patients. Results for CRT-D and CRT-P were similar to ICD both in the United States and in Europe.

**Table 2:** Comparison of survey findings and HRS consensus statement–recommended follow-up practices.

Level	HRS consensus statement	Survey finding
1A	In patients with CIEDs, RM is recommended as part of the standard of care.	United States: 96% RM adoption for PM/ICD
		Europe: 54%/75% RM adoption for PM/ICD
3: No benefit	In patients with ILRs on RM with consistent connectivity, in office visits are not indicated for routine patient care.	43% report scheduling routine office visits for ICM patient
2A	In patients with PMs on RM with consistent and continuous connectivity, and in the absence of recent alerts or other cardiac comorbidity, it is reasonable to schedule in-clinic visits every 24 mo.	7% report every 24 mo; 90% report yearly or twice per year
2A	In patients with ICDs on RM with consistent and continuous connectivity, and in the absence of recent alerts or other cardiac comorbidity, it is reasonable to schedule in-clinic visits every 24 mo.	5% report every 24 mo; 91% report yearly or twice per year
1B	For the care of patients with CIEDs on RM, it is recommended that health care payers adopt adequate reimbursement for RM that is tailored to regional health system care patterns and facilitates sustainable and cost-effective CIED follow-up care.	For 70%–88% scheduled transmissions in the United States the frequency is aligned with reimbursement rules
1B	For the care of patients with CIEDs on RM, a team-based organizational model with formal policies, procedures, and clear definitions of the roles and responsibilities of qualified staff is recommended to optimize all related RM tasks.	Nurses and technicians handle the majority of tasks whereas the Consensus suggests distribution across more staff roles
1B	For the care of patients with CIEDs on RM, it is recommended that there is adequate dedicated time to perform all RM tasks, including scheduled and nonscheduled transmission, patient follow-up, and administrative tasks.	42% of respondents reported somewhat or very insufficient staffing
2A	For the care of patients with CIEDs on RM, it is reasonable to use third-party resources to alleviate RM workload for staff.	11%-21% of clinic report outsourcing tasks considered most burdensome

CIED, cardiac implantable electronic device; HRS, Heart Rhythm Society; ICM, insertable cardiac monitor; ILR, implantable loop recorder; PM, pacemaker; RM, remote monitoring.

**Figure 2:**
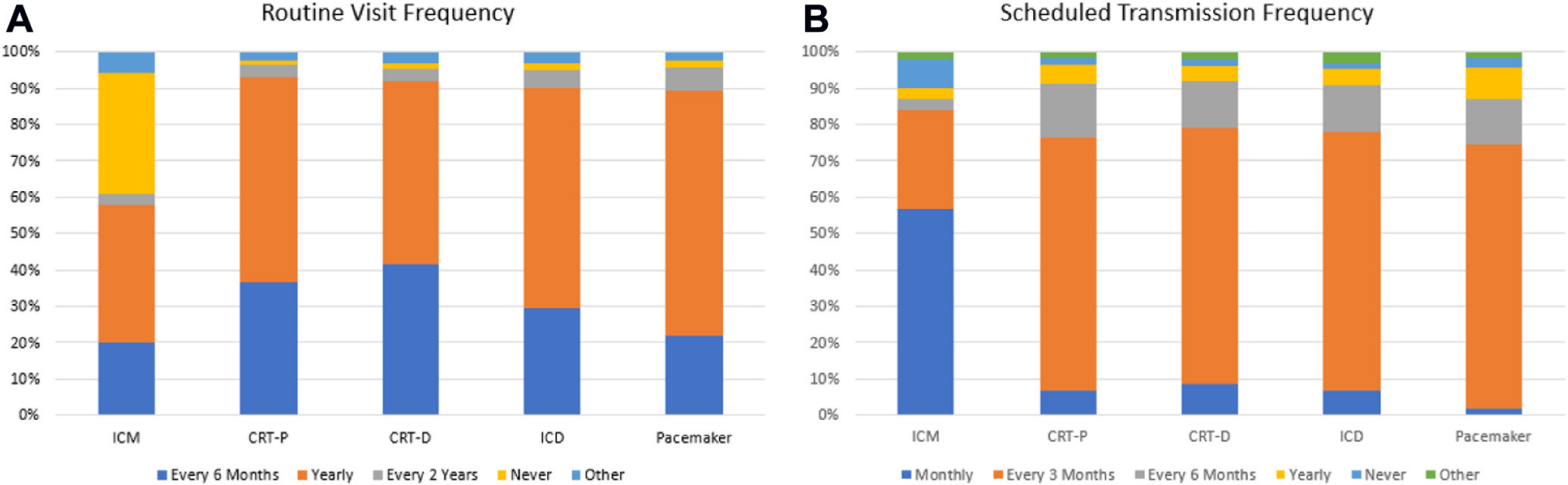
Frequency of routine office visits (A) and scheduled remote transmissions (B) for clinics under hybrid follow-up, by medical device. CRT-D, cardiac resynchronization therapy defibrillator; CRT-P, cardiac resynchronization therapy pacemaker; ICD, implantable cardioverter-defibrillator; ICM, insertable cardiac monitor.

Among respondents who reported using a hybrid management approach, the frequency of routine office visits and scheduled transmissions is presented by device type in Figure 2. While some differences were observed across device types, the most common routine office visit frequency was “once a year” for every device type, ranging from 44% for ICMs to 66% for pacemakers. For pacemaker, CRT-P, CRT-D, and ICD patients, the second most common office visit frequency was “every 6 months,” while the second most common office visit frequency for ICM patients was “never.”

The predominant frequency of scheduled remote transmissions was “every 3 months” for patients with pacemaker, CRT-P, CRT-D, and ICD devices, while “monthly” was the predominant practice for patients with ICMs. However, subanalyses revealed more homogenous follow-up in the United States than in Europe. For patients with a pacemaker, 88% (n = 172) of respondents from the United States reported that transmissions were scheduled every 3 months, while European respondents were split between every 3 months (26% [n = 7]), every 6 months (30% [n = 8]), and yearly (30% [n = 8]). Similarly, for patients with ICMs, 70% (n = 81) of respondents from the United States reported monthly scheduled transmissions, while European respondents were split between never (23% [n = 6]), monthly (19% [n = 5]), and every 3 months (35% [n = 9]).

### Staffing roles

**Figure 3:**
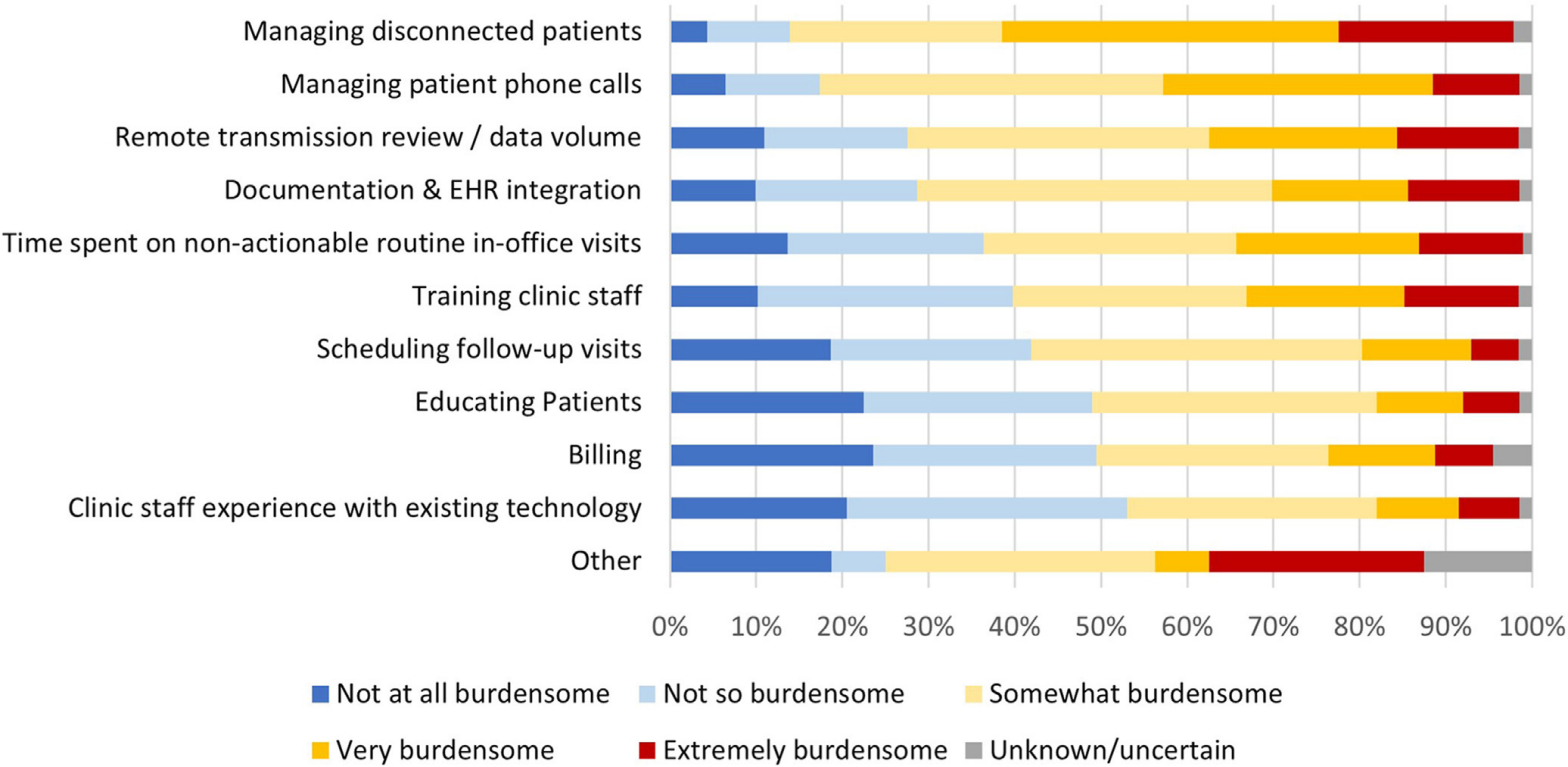
Perceived staffing burden of clinic tasks. EHR, electronic health record.

**Figure 4:**
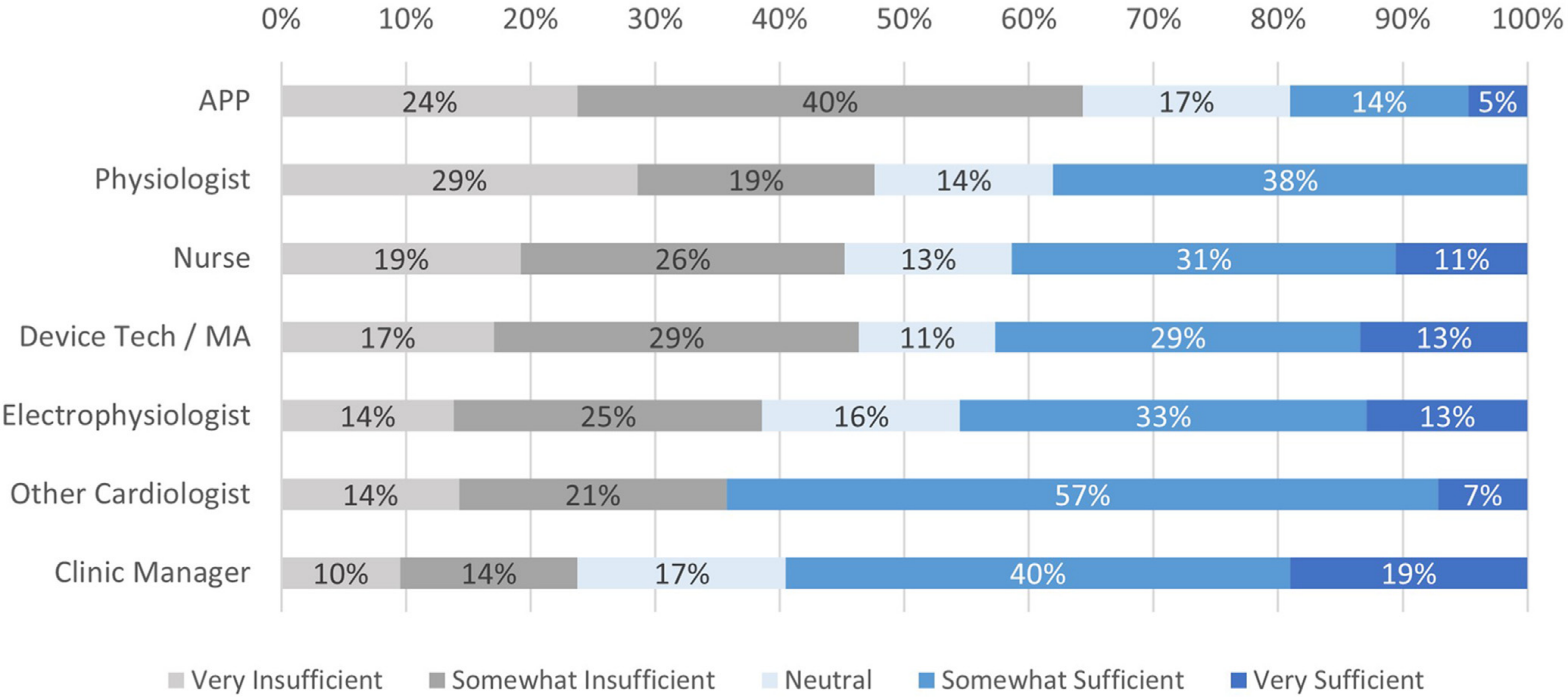
Perceptions on staff sufficiency, by medical specialty. APP, advanced practice provider; MA, medical assistant.

Respondents were queried on the staff type that completes each task pertaining to in-office tasks, RM, and other patient management tasks, and these results are presented in Supplemental Figures 2, 3, and 4. The perceived burden of clinic tasks varied (Figure 3), with remote transmission review, managing disconnected patients, managing patient phone calls, and documentation considered as the most burdensome tasks. Only 10% to 20% of respondents reported outsourcing in-clinic tasks but perceived that high-burden tasks were more likely to be outsourced or considered for outsourcing (Supplemental Figure 5). In terms of staff sufficiency, 42% (n = 180) of respondents indicated that their clinic had somewhat to very insufficient staffing. In particular, advanced practice providers more frequently reported somewhat to very insufficient staffing (Figure 4). No apparent differences were observed between respondents from hospital-based or independent clinics (data not shown).

### Perceptions on the efficiency of care

**Figure 5:**
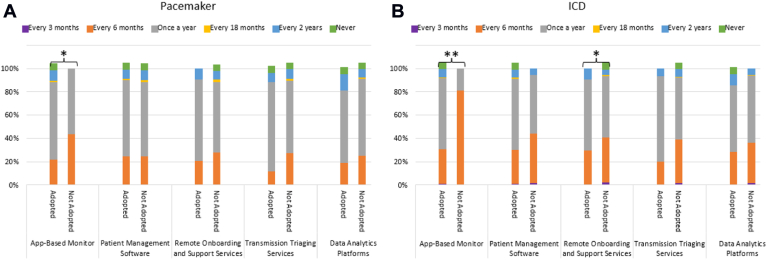
Routine office visit frequency by solution adoption and device type. ICD, implantable cardioverter-defibrillator.

Overall, 60% (n = 258) of respondents rated their clinic as somewhat to very efficient, whereas 25% (n = 107) rated their clinic as somewhat to very inefficient. The majority of office visits were considered routine by most respondents for every device type, and a majority of respondents reported that only few routine office visits led to any medical action (Supplemental Figure 6). Clinics adopting smartphone app-based monitors reported fewer routine office visits for patients with pacemakers (*P* = .03) and ICDs (*P* < .0001), while clinics adopting remote onboarding and support services reported fewer routine office visits for patients with ICDs (*P* = .02) (Figure 5).

Only a minority (n = 60 [29%]) of respondents reported their clinic to be tracking performance, in which case most frequent metrics included number of patients, number of transmissions and alerts, and number of routine office visits (Supplemental Figure 7).

## Discussion

The literature detailing CIED patient management practices is limited. Recently, results from another survey—also disseminated among the HRS network—were published, investigating the use of RM and organization of CIED clinics to study the determinants of RM utilization.^6^ The present international survey study builds on this work by detailing both in-person and remote patient management practices, as well as by examining staffing roles and perceptions. Ultimately, a robust response was received with a total of 471 survey respondents, including broad representation across different provider types, levels of experience, and geographic regions. However, the survey primarily included respondents from the United States and Europe.

### Reported device management practices highlight opportunities for improvement

The 2023 HRS/EHRA/APHRS/LAHRS expert consensus statement on practical management of the remote device clinic^2^ details the increasing complexity and resource strain in remote device management and recommends evidence-based practices to optimize both patient outcomes and device clinic efficiency. The present survey—disseminated 4 to 7 months following publication of the 2023 consensus statement—describes the baseline state of device clinic practices and highlights opportunities for targeted quality improvement initiatives to align device clinic practice with new guidelines. Table 2 summarizes the recommended follow-up practices from the 2023 consensus statement, with the predominant follow-up practices reported in the survey. While meaningful gaps between the consensus statement recommendations and current practice were observed, guideline implementation is likely to take time, and it was encouraging that a majority (71%) reported being aware of the consensus statement.

While reported RM adoption—a 1A recommendation in the consensus statement—in the United States was very high for all device types (94%–96%), adoption lagged in Europe (54%–75%), particularly for pacemaker patients. Given the significant workload to effectively perform RM,^3^ this discrepancy between the United States and Europe may pertain to specific and more consistent reimbursement for RM in the United States. Indeed, a correlation was observed between frequency of scheduled transmissions and Current Procedural Terminology rules for remote monitoring in the United States (pacemaker, ICD, and CRT devices on a quarterly basis, ICM on a monthly basis), which on one side homogenizes follow-up practices and ensures uptake of RM but may create barriers to the adoption of more efficient follow-up practices for which proper funding incentives are currently lacking. For instance, the 2023 consensus suggests that “Alert-based RM…has the potential to replace structured intermittent device follow-up (whether in-person or remote).^2^ This could minimize low-value effort, optimize clinic visits for actionable events, and decrease health care costs.” However, the correlation between reimbursement structure and care practices observed in the present survey suggests that this vision may only be realized if economic incentives are aligned for clinics.

In Europe, reimbursement is more heterogeneous, sometimes not even clearly targeted toward RM, which is likely reflected in the results of this survey by a broad range of answers with respect to the frequency of scheduled transmissions and by the lower RM adoption reported. Workflow optimization leveraging the continuous nature of remote follow-up (eg, through alert-based RM), likely requires a different funding model not based on a defined frequency but still ensuring appropriate funding for performing all RM-related tasks. In some European countries, such models have been recently introduced and their impact will have to be evaluated.^7^^,^^8^

With respect to the frequency of routine office visits, survey results highlighted a gap between the new recommendation and current practice. The 2023 consensus statement states that for ICD and pacemaker patients “on RM with consistent and continuous connectivity, and in the absence of recent alerts or other cardiac comorbidity, it is reasonable to schedule in-person visits every 24 months”.^2^ Our survey found that approximately 90% of respondents reported scheduling routine office visits for the average pacemaker or ICD patient either once or twice per year. Similarly, the consensus statement does not recommend scheduling routine office visits for patients with ICMs with consistent and continuous RM. Nonetheless, 43% of respondents reported scheduling routine office visits for this population. This disparity between consensus recommendations and real-world practice may be due to challenges in achieving consistent and continuous RM at this time, status quo bias, or patient preference. Given the low actionability of routine office visits—reported both in this survey, as well as numerous other studies^9^, ^10^, ^11^, ^12^—and the significant workload associated with these visits,^3^ this consensus recommendation is a meaningful target to streamline clinic efficiency, if consistent and continuous connectivity can be achieved through optimal workflows and supporting technology.

Clinic staffing roles is a key area covered in the 2023 consensus statement. Ensuring that clinic staff dedicate most of their time to tasks commiserate with their expertise is not only important for staff retention, but also economically efficient economically for clinics. The 2023 consensus statement recommends that “ancillary staff” perform tasks pertaining to “appointments, connectivity, and answering patient questions.” The present survey found that nurses and device techs were most often the staff type reported to answer patient questions and manage connectivity issues, representing an opportunity for improvement. However, it is notable that only 1% (n = 6) of respondents were administrative assistants in the present survey. Thus, further research is needed on staffing roles.

### Clinic performance assessment was reported to be uncommon

A small minority of respondents (29%) reported their clinic to be engaged in ongoing performance assessment. Moreover, clinics tracking performance metrics predominantly tracked measures of productivity (eg, number of transmissions reviewed, visits), rather than measures of quality. To effectively implement the recommendations of the 2023 HRS consensus statement, ongoing quality assessment is likely to be crucial. Specific quality metrics to be considered in future initiatives are RM compliance, percent of patients connected and transmitting to the clinic, transmission review backlog, and time to reading remote transmissions. Consistent performance on these indicators will enable transitioning from routine, prescheduled care for all patients (ie, more frequent office visits) to more targeted, prioritized care for patients at the time they need it.

### Limitations

This survey study had several limitations to note. First, the survey was primarily distributed through HRS channels, including at the HRX conference and through e-mail blasts to the HRS membership. It may be that the HRS membership, for instance, is more adherent to modern follow-up practices such as RM. Second, due to the collection of anonymous responses, it was not possible to associate multiple responses received from the same institution. Second, the average completion time for the survey was approximately 30 minutes, which led to significant attrition in survey completion with only 175 (37%) completing every question. The analysis opted to analyze all data available for each question, rather than excluding noncomplete answers. Finally, there were very few responses received outside of the United States and Europe, rendering other region-specific analyses impossible.
