# Supplementary material for: Current practices in managing patients with cardiac implantable electronic devices: Results of an international survey
Source: Heart Rhythm O2. 2025 Mar 5;6(6):781–8. doi: 10.1016/j.hroo.2025.02.019 (PMC12287964; doi:10.1016/j.hroo.2025.02.019)
Supplement: Supplementary Figures and Table [file mmc1.docx]

**SUPPLEMENTARY FIGURES AND TABLES**

**Supplementary Figure 1a:** 2023 Consensus Statement awareness by number of patients managed, stratified by device type

**Supplementary Figure 1b:** 2023 Consensus Statement awareness by staffing type

**Supplementary Table 1:** **Follow-up approaches, by device type and by region**

|  | Both in-clinic and remotely | In-clinic management only | Not managing device type | Remote management only | Unknown |
| --- | --- | --- | --- | --- | --- |
| **Pacemaker, n (%)** | | | | | |
| Total | 258 (78) | 51 (15) | 5 (2) | 11 (3) | 7 (2) |
| Europe | 30 (49) | 23 (38) | 3 (5) | 3 (5) | 2 (3) |
| United States of America | 204 (93) | 5 (2) | 0 (0) | 6 (3) | 5 (2) |
| Other | 24 (47) | 23 (45) | 2 (4) | 2 (4) | 0 (0) |
| **Implantable Cardioverter Defibrillator, n (%)** | | | | | |
| Total | 274 (83) | 32 (10) | 8 (2) | 10 (3) | 8 (2) |
| Europe | 43 (70) | 11 (18) | 2 (3) | 3 (5) | 2 (3) |
| United States of America | 206 (94) | 1 (<1) | 3 (1) | 5 (2) | 5 (2) |
| Other | 25 (49) | 20 (39) | 3 (6) | 2 (4) | 1 (2) |
| **Implantable Cardiac Monitor / Implantable Loop Recorder, n (%)** | | | | | |
| Total | 167 (50) | 22 (7) | 8 (2) | 122 (37) | 13 (4) |
| Europe | 27 (44) | 5 (8) | 1 (2) | 26 (43) | 2 (3) |
| United States of America | 121 (55) | 5 (2) | 2 (1) | 85 (39) | 7 (3) |
| Other | 19 (37) | 12 (24) | 5 (10) | 11 (22) | 4 (8) |
| **Cardiac Resynchronization Therapy Defibrillator, n (%)** | | | | | |
| Total | 276 (83) | 30 (9) | 9 (3) | 7 (2) | 10 (3) |
| Europe | 45 (74) | 10 (16) | 2 (3) | 2 (3) | 2 (3) |
| United States of America | 203 (92) | 2 (1) | 5 (2) | 4 (2) | 6 (3) |
| Other | 28 (55) | 18 (35) | 2 (4) | 1 (2) | 2 (4) |
| **Cardiac Resynchronization Therapy Pacemaker, n (%)** | | | | | |
| Total | 278 (84) | 32 (10) | 6 (2) | 6 (2) | 10 (3) |
| Europe | 43 (70) | 12 (20) | 2 (3) | 2 (3) | 2 (3) |
| United States of America | 207 (94) | 2 (1) | 2 (1) | 3 (1) | 6 (3) |
| Other | 28 (55) | 18 (35) | 2 (4) | 1 (2) | 2 (4) |

**Supplementary Figure 2.** Reported staffing roles for tasks related to office visits

**Supplementary Figure 3.** Reported staffing roles for tasks related to review of remote transmissions

**Supplementary Figure 4.** Reported staffing roles for tasks related to connectivity, education, and remote monitoring enrolment

**Supplementary Figure 5.** Outsourcing adoption by clinic task

**Supplementary Figure 6.** Proportion of office visits considered routine and leading to medical action, by medical device


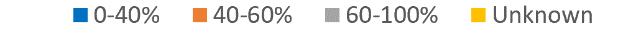

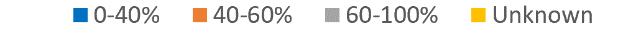


**Supplementary Figure 7.** Number of respondents tracking each clinic performance metric
